# Chimera Spectrum Diagnostics for Peptides Using Two-Dimensional Partial Covariance Mass Spectrometry

**DOI:** 10.3390/molecules26123728

**Published:** 2021-06-18

**Authors:** Taran Driver, Nikhil Bachhawat, Leszek J. Frasinski, Jonathan P. Marangos, Vitali Averbukh, Marina Edelson-Averbukh

**Affiliations:** Department of Physics, Imperial College London, Prince Consort Road, London SW7 2AZ, UK; tdriver@stanford.edu (T.D.); nikhil.bachhawat@stonybrook.edu (N.B.); l.j.frasinski@imperial.ac.uk (L.J.F.); j.marangos@imperial.ac.uk (J.P.M.)

**Keywords:** tandem mass spectrometry, chimera spectra, two-dimensional partial covariance mass spectrometry

## Abstract

The rate of successful identification of peptide sequences by tandem mass spectrometry (MS/MS) is adversely affected by the common occurrence of co-isolation and co-fragmentation of two or more isobaric or isomeric parent ions. This results in so-called `chimera spectra’, which feature peaks of the fragment ions from more than a single precursor ion. The totality of the fragment ion peaks in chimera spectra cannot be assigned to a single peptide sequence, which contradicts a fundamental assumption of the standard automated MS/MS spectra analysis tools, such as protein database search engines. This calls for a diagnostic method able to identify chimera spectra to single out the cases where this assumption is not valid. Here, we demonstrate that, within the recently developed two-dimensional partial covariance mass spectrometry (2D-PC-MS), it is possible to reliably identify chimera spectra directly from the two-dimensional fragment ion spectrum, irrespective of whether the co-isolated peptide ions are isobaric up to a finite mass accuracy or isomeric. We introduce ‘3-57 chimera tag’ technique for chimera spectrum diagnostics based on 2D-PC-MS and perform numerical simulations to examine its efficiency. We experimentally demonstrate the detection of a mixture of two isomeric parent ions, even under conditions when one isomeric peptide is at one five-hundredth of the molar concentration of the second isomer.

## 1. Introduction

Tandem mass spectrometry (MS/MS) has become the method of choice for the analysis of protein molecules, following major technological advancements in sample preparation, measurement hardware and data analysis techniques over the past several decades [1]. One of the challenges still faced by the technique is the pervasive problem of so-called chimera spectra, where two or more parent ions are co-selected for fragmentation in the MS/MS workflow [2]. This is estimated to occur for up to ∼50% of all fragment ion spectra in a standard HPLC-MS run [2,3]. Chimera spectra hinder the automated sequence-to-spectrum assignment, for example, via reduction of the scores of the correct peptide sequences in the presence of contaminant fragment ions of a co-fragmented sequence [2]. At the same time, a reliable recognition of the chimera spectra holds potential for revealing and correctly assigning multiple co-fragmented sequences [4].

### 1.1. Identification of Chimera Spectra—State of the Art

Identification of the chimera spectra stemming from co-fragmenting approximately isobaric sequences can be achieved using a high quality, high mass resolution MS analysis of the precursor ions prior to fragmentation, which forms the basis of multiplexed tandem MS [5]. On lower resolution instruments, chimera spectra can be identified using an improved estimation of the precursor mass [3]. Such approaches, however, are fundamentally limited by the requirement of a measurable m/z separation between the multiple co-isolated parent ions, precluding identification of the chimera spectra for co-isolated isomeric parent ions. Therefore, comprehensive discovery of chimera spectra has to rely on the analysis of the fragment spectra. For example, within the CharmeRT algorithm [6], one attempts to detect a series of high-abundance fragments that could be assigned to a particular sequence using a database search, then remove all the peaks that could be assigned to the candidate sequence and analyze the remaining signals for the presence of the co-fragmented peptides. Other approaches to chimera spectra identification recognize the key importance of the complementary fragment ion pairs to revealing the presence of multiple co-fragmented sequences. In Reference [7], Kryuchkov et al. separated series of identified complementary fragment ions on the basis of their calculated parent ion m/z ratio, still leaving the co-fragmentation of isomers unaddressed. Later, the same group has developed a tagging approach based on the simultaneous detection of the N-terminal b${}_{2}$ and a${}_{2}$ ions, as well as the complementary y${}_{n-2}$ fragments in HCD spectra [8]. The presence of two (or more) such ion series is indicative of the co-fragmentation of multiple precursors, whether isomeric or approximately isobaric. Such approaches crucially depend on the interpretation of certain peaks in a one-dimensional tandem MS spectrum as complementary pairs, typically by the available database search or de novo sequencing software. These identifications are assumption-based and are prone to false-positives and false-negatives, as internal ions can be accidentally isobaric with the supposed terminal fragments, while true terminal fragments may appear at insufficient intensity for the standard software to recognize them as significant.

The recently introduced method of two-dimensional partial covariance mass spectrometry (2D-PC-MS) [9] (also see Reference [10]) allows unambiguous identification of the complementary ion pairs based on the geometrical position of their correlation signals on the 2D map, therefore providing, as we show here, a direct route to diagnostics of the chimera spectra. Due to the assumption-free nature of the complementary pair identification in 2D-PC-MS, the new two-dimensional chimera diagnostics is free of any limitations of the existing 1D techniques, such as requirement of high mass resolution, requirement of the detection of specific fragment (“tags”), or the inability to resolve co-fragmenting isomers. In what follows, we briefly describe 2D-PC-MS, present the new 2D chimera diagnostics method, demonstrate it by applying to mixtures of co-fragmented isomeric peptides and investigate the reliability of the method by numerical simulations.

### 1.2. Two-Dimensional Partial Covariance Mass Spectrometry

Two-dimensional partial covariance mass spectrometry (2D-PC-MS) is a new kind of two-dimensional MS, based entirely on fragment-fragment correlations [9,10]. By mapping the fluctuations in fragment ion abundances across a series of repeated fragment mass spectra, 2D-PC-MS identifies fragment ions born in the same or consecutive decomposition reactions of the same parent molecular ion. This is in sharp contrast to the well familiar two-dimensional Fourier transform ion cyclotron resonance (2D FT-ICR) mass spectrometry, which identifies correlations between a *parent* ion and a fragment ion [11]. A significant fraction of the fragment-fragment correlations revealed by 2D-PC-MS have been shown to be much more structurally specific than the individual fragment ion signals of 1D MS/MS [9]. The 2D spectral information accessible via 2D-PC-MS has been used to solve the long-standing problem of resolving diacetylated isomers of histone H4 peptides [12], and its application to top-down measurements of intact proteins provides a straightforward means to deconvolve the overlapping fragment ion peaks of co-isolated intact proteins [13].

2D-PC-MS is based on measurement of the self-correcting partial covariance between the signals of the fragment ions X and Y across a large number of individual fragment mass spectra (e.g., microscans of a linear ion trap mass spectrometer):

(1) $$pCov\left(X,Y;\mathrm{TIC}\right)=Cov\left(X,Y\right)-\frac{Cov(X,\mathrm{TIC})Cov(Y,\mathrm{TIC})}{Cov(\mathrm{TIC},\mathrm{TIC})}\;,$$

where Cov(X,Y) is the simple covariance between the signals of X and Y, Cov(X,Y)=〈XY〉−〈X〉〈Y〉, 〈…〉 denoting the averaging over the individual fragment spectra. TIC is the total ion count within a given single fragment spectrum, i.e., the partial covariance parameter derived from the spectrum itself [9]. The fragment ions X and Y produced in the same or in consecutive decomposition processes are characterized by positive peaks of self-correcting partial covariance (1) on the 2D-PC-MS map, symmetrical with respect to the $m_{X}/z_{X}=m_{Y}/z_{Y}$ diagonal. If X and Y are two complementary fragments, their masses and charges are related to the mass, ${M_{r}}_{P}$ and charge $Z_{P}$ of the parent peptide ion (P) by mass and charge conservation laws:

(2) $$\left(m_{X}/z_{X}\right)\times z_{X}+\left(m_{Y}/z_{Y}\right)\times z_{Y}={M_{r}}_{P}\;,\;\;\;z_{X}+z_{Y}=Z_{P}.$$

therefore, 2D-PC-MS signals of correlations between complementary fragments lie on straight lines on a 2D-PC-MS map with slopes $-z_{X}/z_{Y}$, called primary mass conservation lines. Correlations between a terminal fragment and a complementary ion which has undergone a neutral loss are offset from the primary mass conservation lines by the mass of the lost neutral molecule divided by the charge of its parent fragment. Finally, fragment correlations resulting from two peptide bond dissociations, for example, those between a terminal and an internal ion, form a manifold of scattered signals on the 2D-PC-MS map.

Efficient discrimination between true low-intensity 2D-PC-MS correlations (X,Y) and statistical noise stemming from the finite number of scans used for the self- correcting partial covariance in Equation (1) is achieved using the peak score, S(X,Y),

(3) $$S\left(X,Y\right)=\frac{V[pCov(X,Y;\mathrm{TIC})]}{\sigma (V)}\;,$$

where V[pCov(X,Y;TIC)] is the 2D-PC-MS peak volume, and σ(V) is the standard deviation of the peak volume under jackknife resampling. A proteomic database search engine relying exclusively on 2D-PC-MS correlations scored using Equation (3) has been shown to outperform a state-of-the-art 1D MS/MS search engine in the challenging case of chimera spectra formed by a mixture of palindromic (and therefore fully isomeric) sequences [14].

## 2. Results

### 2.1. Principles of Chimera Spectra Detection within 2D-PC-MS

As a direct consequence of mass and charge conservation in the course of the peptide decomposition, the 2D-PC-MS correlation signals of the pairs of complementary terminal fragment ions (e.g., b${}_{i}$/y${}_{n-i}$ fragments in the collision-induced dissociation (CID) measurements [15]) can be easily identified as lying on the primary mass conservation lines, see Equation (2). The equations of all the possible primary mass conservation lines for a parent ion of molecular mass ${M_{r}}_{P}$ and charge $Z_{P}$ can be expressed as:

(4) $$y=-\frac{z_{1}}{z_{2}}\times x+\frac{{M_{r}}_{P}}{z_{2}},$$

where the complementary fragment ion charges $z_{1}$ and $z_{2}$ are constrained by $z_{2}\geq z_{1}$ (to avoid double counting) and $z_{1}+z_{2}=Z_{P}$. Therefore, provided knowledge of the charge state and m/z of a parent ion, it is possible to define all the primary mass conservation lines for its decomposition products a priori, without any assumption on the peptide sequence or the type of the decomposition products (e.g., b/y vs. c/z). As a result, all the measured pairs of complementary ions can be read directly from the 2D-PC-MS map, assumption-free. This unique feature of the 2D-PC-MS is key to the proposed method of the chimera diagnostics.

In what follows, we shall distinguish between three distinct scenarios leading to chimera spectra:

**Scenario 1**  co-fragmented parent ions have different charges, but arbitrarily close m/z;
**Scenario 2**  co-fragmented parent ions have the same charge, while their masses are close, but distinguishable within the mass resolution of the MS equipment; and
**Scenario 3**  co-fragmented parent ions have the same charge and either indistinguishable (within the mass resolution of the equipment) or exactly the same masses.

Under **Scenario 1**, i.e., if the co-isolated parent ions have a different charge state, Equation (4) dictates that their fragment ions will fall on two different sets of the primary mass conservation lines, characterized by different $-z_{1}/z_{2}$ slopes and ${M_{r}}_{P}/z_{2}$ y-axis intercepts. As demonstrated in Reference [13], this enables immediate identification of the chimera spectrum, as well as its in silico deconvolution. Under **Scenario 2**, i.e., if the co-isolated parent ions have the same charge state, but the m/z tolerance of the fragment ion detector is sufficient to resolve the difference in the sums of their masses, the complementary ions from the different co-isolated parents are separated by virtue of falling on primary mass conservation lines with the same slopes, but different y-axis intercepts, ${M_{r}}_{P}/z_{2}$, see Equation (4). It is only left, therefore, to consider the most challenging **Scenario 3**, under which the 2D-PC-MS correlations between the complementary fragment ions of the co-fragmented peptides will fall on exactly the same primary mass conservation lines (for isomers) or on the primary conservation lines indistinguishable within the instrumental mass resolution. This scenario is considered in detail below.

Without loss of generality, consider the complementary fragment ions formed in CID, i.e., b${}_{i}$/y${}_{n-i}$. Within this series of complementary pairs, each successive b-ion or y-ion is separated from the next one of the same type by the mass of the next amino acid residue. The amino acid residue of the lowest mass (57 Da) is glycine, where the functional R-group is a single H atom. Looking at the 2D-PC-MS correlation signals of the pairs of complementary fragments on all the primary mass conservation lines, we do not know whether any particular fragment within a pair is of b- or y-type. Therefore, if three or more fragment ions forming correlations which are found on a mass conservation diagonal within a mass interval of 57 Da or less, there must exist at least two b-ions or y-ions in the fragment spectrum which are within less than 57 Da mass of each other. In this case, either all three ions are of the same type, or one is a b-(y-)ion, and the other two are y-(b-)ions. Since the minimum mass by which two consecutive b- or y-ions from the same sequence can be separated is the mass of glycine residue (57 Da), two b- or y-ions separated by less than 57 Da cannot come from the same parent sequence; therefore, we observe a chimera spectrum. In this line of reasoning, we take into account that the charge states of the fragments are known, either via isotopic envelope measurement, or (within 2D-PC-MS) via the measurement of the slope of the primary mass conservation line to which their correlation belongs, as was done in Reference [13].

On the basis of the above, we define a 2D-PC-MS chimera `tag’ as a single set of 3 complementary fragment ions of mass within 57 Da of each other (`3-57 chimera tag’). It is possible for a chimera spectrum to contain multiple such tags (see Section 2.3); however, even a single chimera tag is sufficient to identify co-fragmentation of multiple parent ions with mathematical certainty, purely on the basis of the geometrical positions of the fragment-fragment correlations on the 2D-PC-MS map, prior to any attempt of the sequence assignment. It is important to point out that the 3-57 Da chimera tag is straightforwardly applicable only when the MS/MS spectrum features complementary ion pairs of a single type only, such as b${}_{i}$/y${}_{n-i}$ under positive ion mode CID. This may not always be the case in ECD, where b/y complementary fragments can be observed (see, e.g., Reference [16]), despite the general dominance of the c/z fragment ions [17], as well as in the certain types of sequences under negative ion CID, where formation of c/z complementary pairs is possible alongside the dominant backbone fragmentation leading to the b/y ions [18]. In such uncommon cases, 3-57 chimera tags featuring fragments with certain characteristic mass differences (e.g., b-c mass difference of 15 Da) should be disregarded as not necessarily informative of multiple peptide co-fragmentation.

### 2.2. Experimental Demonstration of 3-57 Tags

**Figure 1:**
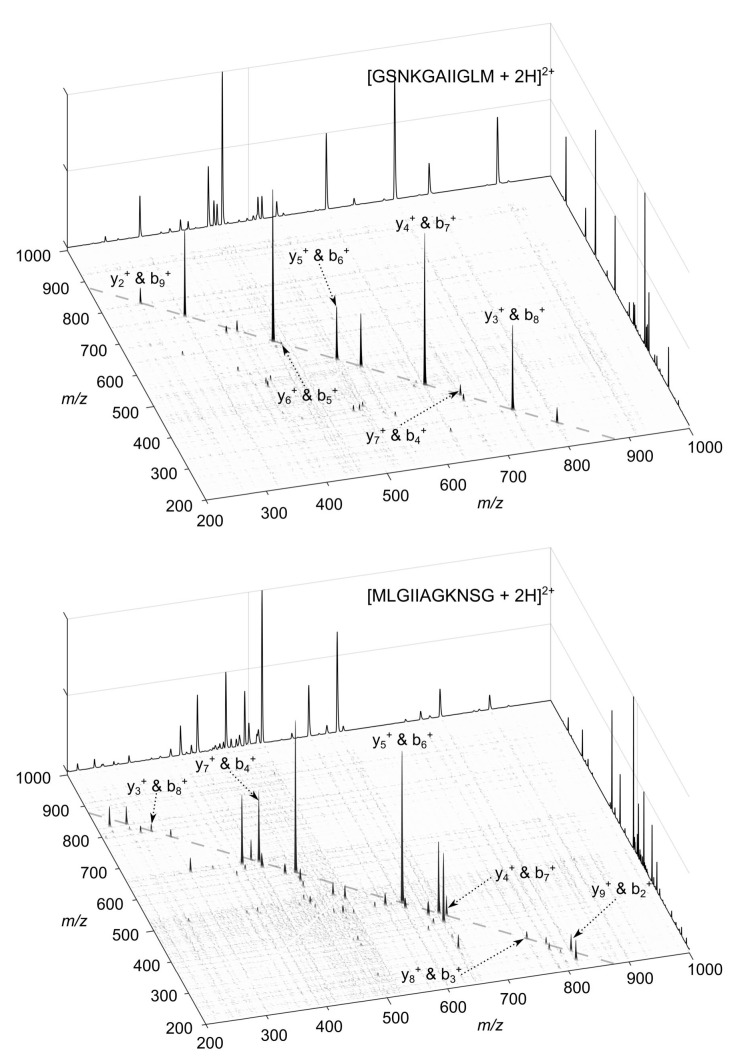
Three-dimensional view of the 2D-PC-MS maps of [GSNKGAIIGLM+2H]^2+^ (**I1**^2+^, **top**) and [MLGIIAGKNSG+2H]^2+^ (**I2**^2+^, **bottom**). The standard 1D tandem mass spectra are appended to the x- and y-axes for visual clarity. The single primary mass conservation line, characteristic of doubly charged peptides (see Equation (4)), is shown by the dashed line. Fragment-fragment correlations between the complementary b- and y-fragments lying on the primary mass conservation line are annotated.

We demonstrate the principle of operation of the 2D-PC-MS 3-57 chimera tag experimentally using mixtures of palindromic (i.e., fully isomeric) sequences GSNKGAIIGLM (**I1**) and MLGIIAGKNSG (**I2**). The 3D view of the 2D-PC-MS maps of the pure ions [**I1** + 2H]^2+^ and [**I2** + 2H]^2+^ are shown in Figure 1. The specific choice of the doubly charged states of the peptides in the experimental investigation of the chimera spectra was motivated by the fact that the two-fold chimera spectra of doubtly charged ioan is the most challenging case within the presented approach, with the highest possible probability of false-negative result, see Figure 3. The maps were obtained using a linear ion trap LTQ-XL mass spectrometer (Thermo Fischer Scientific, San Jose, CA, USA ) under CID conditions in the positive ion mode. The details of the experimental procedure are as in Reference [9]; details can also be seen in Materials and Methods.

**Figure 2:**
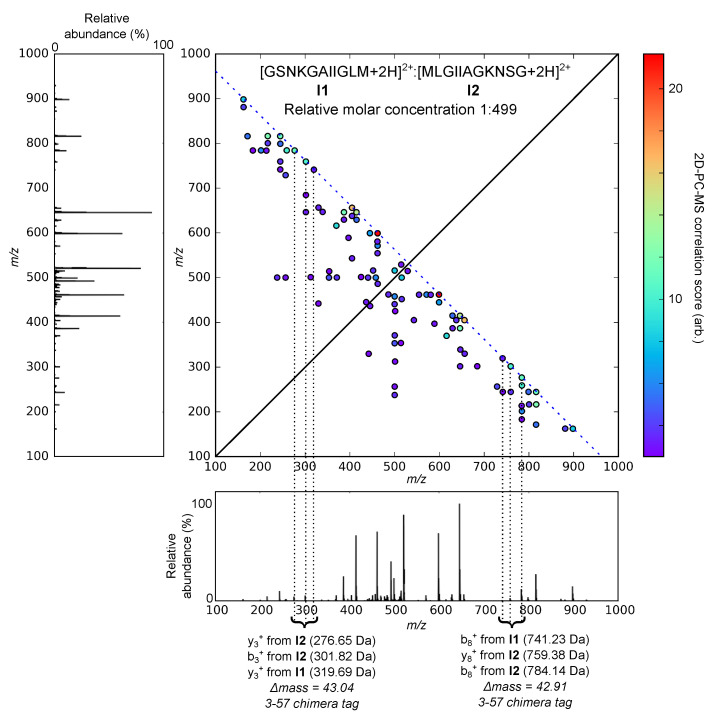
2D-PC-MS map of the chimera spectrum of a mixture of [GSNKGAIIGLM + 2H]^2+^ (**I1**^2+^) and [MLGIIAGKNSG + 2H]^2+^ (**I2**^2+^) at a relative molar concentration of 1:499. The standard 1D tandem mass spectrum of the mixture is appended to the x- and y-axes for visual clarity. The autocorrelation diagonal of the 2D-PC-MS map, with respect to which it is symmetric, is shown by a full line. The autocorrelation signals of each and every fragment with itself are removed for clarity. The single primary mass conservation line, characteristic of doubly charged peptides (see Equation (4)), is shown by the blue dashed line. Fragment-fragment correlations are shown by circles, color-coded according to the values of their 2D-PC-MS correlation scores, Equation (3). 2D-PC-MS correlations of the complementary fragment ions are located on the primary mass conservation line. Two groups of three such correlations are found to lie within Δmass < 57 Da mass range, representing the two 3-57 chimera tags, shown by the vertical dotted lines. The identities of the complementary fragments forming the chimera tag correlations are also shown, although assignment of the complementary ions is not necessary for the 3-57 chimera tag identification; see text for details.

The 2D view of the 2D-PC-MS map of the co-isolated ions [**I1** + 2H]^2+^ and [**I2** + 2H]^2+^ at a relative molar concentration of 1:499 is shown in Figure 2. The complementary fragment correlations among the top 50 2D-PC-MS features ranked according to their correlation score (Equation (3)) were queried for 3-57 chimera tags. In Figure 2, the top 50 2D-PC-MS correlation score-ranked features are plotted for the 1:499 mixture, and the their scores are coded in the color map. The two chimera tags identified even at this extremely low abundance of one of the precursor ions are annotated in Figure 2. The two 3-57 tags clearly indicate that the fragment ion spectrum has resulted from the fragmentation of more than one parent ion. Table 1 shows the growth of the number of the identified 3-57 tags at progressively higher relative concentrations of the isomer **I1**.

### 2.3. Estimation of the False-Negative Rate of Chimera Spectra Detection Using 3-57 Tags within 2D-PC-MS

The TIC-based self-correcting partial covariance (see Equation (1)) has been shown to be false-positive-free [9]. Therefore, the chimera spectra diagnostics using the 2D-PC-MS 3-57 tags presented above is false-positive-free by construction, provided that no double series of complementary ion pairs arise (as, for example, in References [16,18]) or that false tags stemming from such double series can be efficiently rejected on the basis of their characteristic mass difference. However, no chimera diagnostic method, whether 1D MS or 2D-PC-MS based, can be 100% free of false-negatives. Indeed, conditions can always be devised, under which the decompositions of one or more of the co-fragmented peptides will not show strongly enough in the spectrum, therefore suppressing the possible chimera tags. Factors adversely affecting the detection of the chimera tags include relative concentrations of the co-fragmented isomeric or isobaric sequences and sequence-dependent fragmentation efficiency. It is, therefore, left to investigate the rate of false-negatives for the 2D-PC-MS chimera spectra detection based on the 3-57 chimera tags. Here, we perform such an investigation for the positive ion mode CID decompositions by applying numerical simulations to the peptide sequences derived from the UniProt/Swiss-Prot database [19].

The false-negative identifications of chimera spectra using the 2D-PC-MS 3-57 tags could arise only from the lack of formation or detection of the correlations between the complementary ion pairs that could have given rise to the 3-57 tags, such as those in Figure 2. Therefore, the key question for the estimation of the false positive rate is what percentage of the complementary fragment ion correlations is detected by 2D-PC-MS under certain fragmentation conditions. In Reference [9], we performed 2D-PC-MS measurements on 35 various peptide ions under positive ion mode CID (see Table 1 in Reference [9]), which is the most common fragmentation technique in proteomic MS. Statistical analysis of these measurements shows that the obtained 2D-PC-MS maps feature ≈69% of all the theoretically possible b/y complementary pairs for 2+ parent ions and ≈74% of all the theoretically possible b/y complementary ion pairs for the 3+ parent ions. In what follows, we will adopt these detection rates as representative of 2D-PC-MS measurements within the positive ion mode CID.

For the estimation of the false-negative rate of chimera spectra detection by 2D-PC-MS 3-57 chimera tag, we subjected all sequences in the UniProt/Swiss-Prot database [19] to an in silico tryptic digestion (no missed cleavages), omitting the 0.47% of protein sequences with ambiguous amino acid residue coding. From the resultant digestion products, five different sets of 5000 peptide sequences of length between 5 and 15 residues were selected at random. All combinations of such sequences which produce, at a given positive charge state, two parent ions falling within a typical ion trap isolation width of 2.5 Da were identified. From each pool, sets of possible two-fold, three-fold, and four-fold mixtures of peptides with a risk of being co-isolated and co-fragmented were drawn without replacement at random.

Each set of potentially co-isolated sequences was co-fragmented in silico, and the resultant b-ion/y-ion complementary correlations were then assumed to be `measured’ with the experimentally determined average detection probability as reported above, i.e., 69% for doubly charged parent ions and 74% for the triply charged parent ions. To account for experimental inaccuracies, the in silico `measurement’ incorporated a random deviation from the theoretical m/z which was uniformly distributed across $\pm acc_{m/z}$, with $acc_{m/z}$ the specified instrumental m/z accuracy (here, 0.8 Da as in the linear ion trap 2D-PC-MS measurements [9]). For 3+ parents, which generate a 2+/1+ complementary pair, the 2+ fragment was specified to be the longest (in terms of number of residues) of the two, and if the fragments had equal numbers of residues the extra charge was attributed to the y-ion. The resultant b/y complementary pairs measured in silico for each two-, three-, or four-fold chimera spectrum were then queried to identify all 3-57 chimera tags, i.e., all sets of of three or more consecutive complementary ions falling within $57-2\times acc_{m/z}$ of each other for 2+ parent ions, or $57-3\times acc_{m/z}$ for 3+ parent ions. The subtraction of the factors $2\times acc_{m/z}$ or $3\times acc_{m/z}$ accounts for the finite accuracy of the detector. The appearance of a two-, three-, or four-fold chimera spectrum with zero 3-57 tags was then counted as a false negative result.

**Figure 3:**
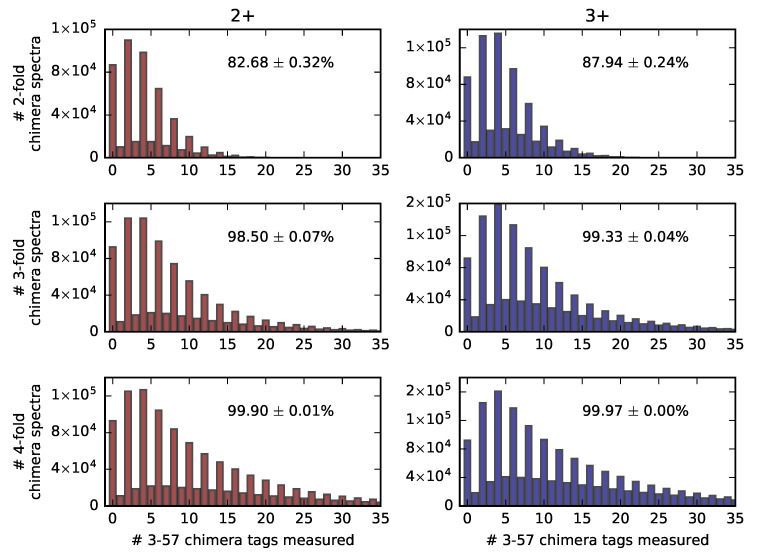
Histograms of the number of chimera tags predicted to be measured by 2D-PC-MS within the positive ion mode CID for two-, three-, and four-fold chimera spectra of 2+ (**left**) and 3+ (**right**) tryptic peptide ions derived from the Swiss-Prot database. The lowest rate of chimera spectra detection is predicted to be approximately 83% (in the two-fold chimera spectra of the doubly charged ions), demonstrating the usefulness of the approach.

For triply charged ions, the numerical simulations involving five different sets of 5000 randomly selected peptides produced the false negative result in 12.06±0.24% of the two-fold chimera spectra, 0.77±0.036% false negatives for the three-fold chimera spectra, and 0.03±0.0022% false negatives for the four-fold chimera spectra. The analogous calculations for the doubly charged parent ions produced false negative rates of 17.32±0.32%, 1.50±0.072% and 0.1±0.015% for the two-, three-, and four-fold chimera spectra, respectively. Figure 3 shows the histograms of the numbers of 3-57 chimera tags estimated to be produced in the positive ion mode CID 2D-PC-MS measurement of two-, three-, and four-fold chimera spectra of doubly and triply charged peptides. In the case of two-fold chimera spectra of the triply charged peptide ions, for example, altogether, $7.29\times 10^{5}$ simulated chimera spectra were used to create the corresponding histogram, resulting in the average number of tags produced across the various simulated two-fold mixtures to be ≈5.

The strong modulation in favor of an even number of detected tags revealed by Figure 3 is readily explained by the symmetry of the 2D-PC-MS map with respect to the autocorrelation diagonal. Indeed, all of the b-(y-)ions contributing to a given 3-57 chimera tag will have complementary y-(b-)ions, whose mass is the difference between the parent molecule and its complementary b-(y-)ion. Given the parent masses of all co-fragmented ions are constrained to be similar (isolation width 2.5 Da in our numerical simulation), and most of the theoretically possible correlations are assumed to be detected (69% or 74% depending on the parent charge state), in the majority of the 3-57 tags the three b- or y-ions within 57 Da mass will have three respective complementary ions which also fall within 57 Da of each other, producing their own, symmetrical chimera tag. This symmetry between the chimera tags produced by the complementary ions can be seen in Figure 2. As a test of our numerical method, we have also simulated complementary ion spectra for 50,000 pure peptides, assuming 100% detection probability for all possible complementary b-ion/y-ion pairs. The simulation produced zero 3-57 chimera tags, as expected of a false-positive-free technique.

## 3. Discussion

Our numerical results show that the 2D-PC-MS chimera diagnostics based on the 3-57 tags introduced in this work is a reliable tool for identifying co-fragmentation of multiple isobaric or isomeric peptides. The appearance of even a single 3-57 tag in the 2D-PC-MS map indicates with mathematical certainty that such a co-fragmentation has indeed taken place (i.e., the technique is false-positive-free), except under the rare conditions of the appearance of multiple complementary fragment ion series in the same spectrum (e.g., both b/y and c/z as in References [16,18]). Even in such unusual cases, the false tags can be discarded using the characteristic mass differences between the two kinds of complementary ions, e.g., 15 Da difference between b${}_{i}$ and c${}_{i}$ fragments. However, absence of the 3-57 tags in the 2D-PC-MS map does not guarantee that the spectrum is not a chimera one, because of incomplete sequence coverage by complementary fragment pairs. In particular, our numerical calculations show that under positive ion mode CID, such false-negative results occur in ≈0.03% to ≈17% of the chimera spectra depending on the charge state and the number of the co-fragmented sequences. These simulations assume equal concentration of the two or more co-fragmented peptides, therefore representing a lower bound of the false-negative rate at an arbitrary concentration ratio. However, while our experimental demonstration on a series of mixtures of palindromic sequences indeed shows that the number of the 3-57 tags decreases with increasing concentration ratio, two symmetrically related 3-57 tags were detected, even in the 1:499 mixture of **I1**^2+^, **I2**^2+^; see Figure 2. The proposed 3-57 tag method is general and can be applied to complementary ion pairs under any fragmentation method. This is in contrast to the previously proposed $b_{2}/a_{2}/y_{n-2}$ chimera tag that requires an HCD measurement [8].

The introduced 3-57 chimera tags can be searched for without any assumption about the presence of such or another sequence in the measured spectrum, as, for example, in Reference [6]. This becomes possible due to the unique feature of 2D-PC-MS that geometrically separates the complementary fragment ion correlations on which the 3-57 tag approach is based, from the rest of the fragment-fragment correlations on the 2D-PC-MS maps. The geometrical positions of the primary conservation lines, where these complementary fragment correlations lie, are fully defined by the mass and charge of the parent ion.This is in sharp contrast to the standard 1D MS/MS, where each individual signal can a priori belong to any terminal or internal ion, and unambiguous identification of two terminal fragments forming a complementary pair is by no means guaranteed. Therefore, while 3-57 tag method can in principle be also applied to chimera spectra diagnostics within 1D MS/MS, it would lose the essential features of the method proposed here. In particular, it would no longer be false-positive-free and would have to rely on the probabilistic assignment of the spectral signals as belonging to complementary fragment ions.

Noteworthy, the presence of the 3-57 chimera tag(s) only provides a yes/no-type indication on the chimera character of the spectrum, i.e., it does not reveal how many different sequences are being co-fragmented. In addition, the 3-57 tags do not currently provide a route to de-convolution of the chimera spectra or even merely to separation of the complementary fragment correlations of two or more co-fragmented sequences [13]. Development of such capabilities within the 2D-PC-MS maps should be subject of future work.

## 4. Materials and Methods

### 4.1. Materials

Water, acetonitrile, and formic acid used for the MS analysis were of Optima LC-MS grade and were purchased from Fisher Scientific Ltd. The peptides **I1** and **I2** were purchased from Bachem AG (Bubendorf, Switzerland).

### 4.2. MS Analysis and Data Processing

2D-PC-MS measurements were performed on a Thermo Fisher Scientific LTQ XL instrument. The instrument required no modification. The MS/MS scans were performed at a scan rate of 125,000 Da/s, with AGC MS^n^ target value of $10^{2}$. The self-correcting partial covariance maps were built according to Equation (1) using 10,000 microscans for the covariance calculations. For the 2D-PC-MS measurements, the synthetic peptides were dissolved in 50% acetonitrile/2% formic acid in water to produce the total concentrations of 10 μM.

The samples were infused into the mass spectrometer via a Harvard Apparatus 11 Plus Single Syringe Pump coupled to a Nanospray II Ion Source (Thermo Fisher Scientific, Waltham, MA, USA) at a flow rate of 3–5 μL/min and spray voltage of 1.8–2.2 kV, using no auxiliary desolvation gas. The temperature of the ion transfer capillary was held constant at 200 °C. The parent ions of interest were fragmented by collision-induced dissociation at normalized collision energies of 35%, with activation time of 30 ms and Mathieu q-value of 0.25.

The 2D-PC-MS data processing was carried out by in-house computer code written in Python (2.7) using numerical routines from the NumPy (http://www.numpy.org/, accesed on 14 June 2021) and SciPy (http://www.scipy.org/, accesed on 14 June 2021) libraries. The software reads in the MS/MS raw data in text file format and calculates the TIC partial covariance (pCov) between each pair of m/z channels in the tandem mass spectra using Equation (1). Another Python code was written for processing the resulting 2D-PC-MS maps to produce the scored lists of fragment ion correlations. This code first determined the features of a 2D-PC-MS map potentially corresponding to true correlation peaks, according to the height of their apices, followed by the calculation of the 2D-PC-MS correlation score using Equation (3).

The code for the simulation of the 3-57 tag false-negative rates, including in silico digestion, in silico fragmentation, 3-57 tag search, was written in the Python (2.7) programming language, making use of the NumPy and SciPy libraries.

## Figures and Tables

**Table 1:** 2D-PC-MS chimera spectrum identification using the 3-57 tags of the doubly charged ions of the mixtures of the isomeric sequences GSNKGAIIGLM (**I1**) and MLGIIAGKNSG (**I2**) across different relative molar concentrations (ratios given in brackets). The number of the 3-57 tags (such as those shown in Figure 2) is seen to increase with increasing **I1** concentration. As expected, no chimera tags are detected in the pure peptide 2D-PC-MS spectra.

Analysed Sample	Chimera Spectrum Identified?	# 3-57 Tags
$[I1\;+\;{2H]}^{2+}:[I2\;+\;{2H]}^{2+}$ (1:499)	Y	2
$[I1\;+\;{2H]}^{2+}:[I2\;+\;{2H]}^{2+}$ (1:99)	Y	6
$[I1\;+\;{2H]}^{2+}:[I2\;+\;{2H]}^{2+}$ (1:19)	Y	10
$[I1\;+\;{2H]}^{2+}:[I2\;+\;{2H]}^{2+}$ (1:9)	Y	10
$[I1\;+\;{2H]}^{2+}:[I2\;+\;{2H]}^{2+}$ (1:1)	Y	14
${\left[\mathrm{GSNKGAIIGLM}\right]}^{2+}$ (I1, pure)	N	0
${\left[\mathrm{MLGIIAGKNSG}\right]}^{2+}$ (I2, pure)	N	0

## Data Availability

The experimenta data for this work is available at Figshare: doi:10.6084/m9.figshare.14788170.
